# Atypical Carboxysome Loci: JEEPs or Junk?

**DOI:** 10.3389/fmicb.2022.872708

**Published:** 2022-05-20

**Authors:** USF Genomics Class 2020, USF Genomics Class 2021, Markus Sutter, Cheryl A. Kerfeld, Kathleen M. Scott

**Affiliations:** ^1^Integrative Biology Department, University of South Florida, Tampa, FL, United States; ^2^MSU-DOE Plant Research Laboratory, Michigan State University, East Lansing, MI, United States; ^3^Environmental Genomics and Systems Biology and Molecular Biophysics and Integrated Bioimaging Divisions, Lawrence Berkeley National Laboratory, Berkeley, CA, United States

**Keywords:** carboxysome, microcompartment, carbonic anhydrase, carbon dioxide fixation, autotroph

## Abstract

Carboxysomes, responsible for a substantial fraction of CO_2_ fixation on Earth, are proteinaceous microcompartments found in many autotrophic members of domain *Bacteria*, primarily from the phyla *Proteobacteria* and *Cyanobacteria*. Carboxysomes facilitate CO_2_ fixation by the Calvin-Benson-Bassham (CBB) cycle, particularly under conditions where the CO_2_ concentration is variable or low, or O_2_ is abundant. These microcompartments are composed of an icosahedral shell containing the enzymes ribulose 1,5-carboxylase/oxygenase (RubisCO) and carbonic anhydrase. They function as part of a CO_2_ concentrating mechanism, in which cells accumulate HCO_3_^−^ in the cytoplasm *via* active transport, HCO_3_^−^ enters the carboxysomes through pores in the carboxysomal shell proteins, and carboxysomal carbonic anhydrase facilitates the conversion of HCO_3_^−^ to CO_2_, which RubisCO fixes. Two forms of carboxysomes have been described: α-carboxysomes and β-carboxysomes, which arose independently from ancestral microcompartments. The α-carboxysomes present in *Proteobacteria* and some *Cyanobacteria* have shells comprised of four types of proteins [CsoS1 hexamers, CsoS4 pentamers, CsoS2 assembly proteins, and α-carboxysomal carbonic anhydrase (CsoSCA)], and contain form IA RubisCO (CbbL and CbbS). In the majority of cases, these components are encoded in the genome near each other in a gene locus, and transcribed together as an operon. Interestingly, genome sequencing has revealed some α-carboxysome loci that are missing genes encoding one or more of these components. Some loci lack the genes encoding RubisCO, others lack a gene encoding carbonic anhydrase, some loci are missing shell protein genes, and in some organisms, genes homologous to those encoding the carboxysome-associated carbonic anhydrase are the only carboxysome-related genes present in the genome. Given that RubisCO, assembly factors, carbonic anhydrase, and shell proteins are all essential for carboxysome function, these absences are quite intriguing. In this review, we provide an overview of the most recent studies of the structural components of carboxysomes, describe the genomic context and taxonomic distribution of atypical carboxysome loci, and propose functions for these variants. We suggest that these atypical loci are JEEPs, which have modified functions based on the presence of Just Enough Essential Parts.

## Introduction

Autotrophic organisms that use the Calvin-Benson-Bassham cycle (CBB) for carbon dioxide fixation must grapple with the catalytic constraints of ribulose 1,5-bisphosphate carboxylase/oxygenase (RubisCO). This enzyme has poor substrate specificity; it catalyzes both the carboxylase reaction of the CBB, as well as a wasteful oxygenase reaction, which results in added energetic expense to regenerate the ribulose 1,5 bisphosphate (RuBP) necessary for the CBB (Tabita, 1999). In addition, RubisCO enzymes have relatively low affinities for CO_2_ (5–250 μM; Tabita, 1999). RubisCO affinities for CO_2_ are particularly low for autotrophic bacteria (25–250 μM; tabulated in Horken and Tabita, 1999). Furthermore, RubisCO is not able to use HCO_3_^−^ (Cooper and Filmer, 1969), the predominant form in the equilibrium between CO_2_ and HCO_3_^−^ at the circumneutral pH typical for cytoplasm.

In order to grow while using CO_2_ as a major carbon source, many autotrophic bacteria using the CBB cycle have CO_2_-concentrating mechanisms (CCMs). CCMs consist of two components: (1) membrane transporters for dissolved inorganic carbon (DIC; = CO_2_ + HCO_3_^−^ + CO_3_^2−^), which generate high concentrations of cytoplasmic HCO_3_^−^, and (2) carboxysomes, which are present in the cytoplasm and facilitate high rates of CO_2_ fixation by RubisCO (reviewed in Price et al., 2009; Long et al., 2016). Carboxysomes are a type of bacterial microcompartment, and consist of a protein shell filled with RubisCO and a trace of carbonic anhydrase activity (reviewed in Kerfeld et al., 2018). Cytoplasmic HCO_3_^−^ enters carboxysomes, where carbonic anhydrase converts some of it to CO_2_, which is then fixed by RubisCO. CO_2_ is prevented from escaping from the carboxysome before fixation because the shell is impermeable to this gas (Dou et al., 2008; Cai et al., 2009). The components of CCMs, including carboxysomes, are often upregulated when autotrophic microorganisms are cultivated under low DIC conditions (Dobrinski et al., 2012; Esparza et al., 2019; Scott et al., 2019).

Two types of carboxysomes (α and β) are currently recognized (reviewed in Cannon et al., 2010; Kerfeld and Melnicki, 2016). Members of *Proteobacteria* and certain marine members of *Cyanobacteria* have α-carboxysomes, while the remaining members of *Cyanobacteria* have β-carboxysomes (Price et al., 2009; Scott et al., 2019). These types can be distinguished by the form of RubisCO they carry (α-carboxysomes carry form IA RubisCO; β-carboxysomes carry form IB RubisCO), as well as differences in carbonic anhydrases, scaffolding proteins, and carboxysome shell components (Kerfeld and Melnicki, 2016).

**Figure 1:**
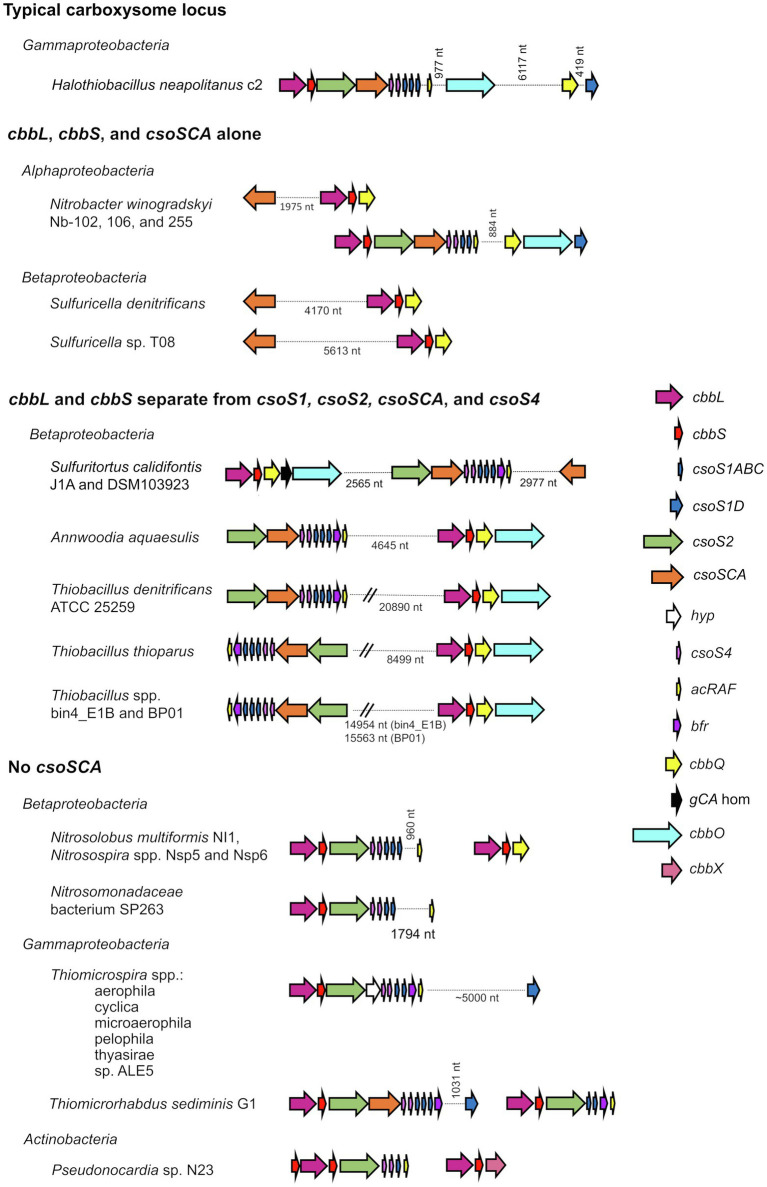
Atypical carboxysome loci. Arrows connected by dotted lines are collocated on the genome, and the distance between them is indicated in nucleotides (nt). *cbbL*, ribulose 1,5-carboxylase/oxygenase (RubisCO) large subunit; *cbbS*, RubisCO small subunit; *csoS1ABC*, hexamer shell proteins; *csoS1D*, pseudohexamer shell protein; *csoS2*, carboxysome assembly protein; *csoSCA*, carboxysomal carbonic anhydrase; *hyp*, hypothetical protein; *csoS4*, pentameric shell protein; *acRAF*, RubisCO assembly factor; *bfr*, bacterioferritin-like protein; *cbbQ*, RubisCO activase; *gCA* hom: gamma carbonic anhydrase homolog; cbbO: adaptor for CbbQ protein; and *cbbX*, RubisCO activase.

The composition of α-carboxysomes from members of phyla *Proteobacteria* and *Cyanobacteria* is mostly conserved (Kinney et al., 2011; Roberts et al., 2012; Sutter et al., 2021). The icosahedral shells of carboxysomes are comprised of (1) hexagonal units, consisting of hexamers of CsoS1 proteins that assemble into single-layers (Tsai et al., 2007), as well as trimers of CsoS1D proteins that assemble into single and double layers (Klein et al., 2009; Roberts et al., 2012), and (2) pentamers of CsoS4 proteins which assemble into pentagonal truncated pyramids and cap the vertices of the icosahedral shells (Tanaka et al., 2008; Cai et al., 2009; Zhao et al., 2019). Hexamers, trimers, and pentamers typically have central pores, which in some cases open and close. The size and charge of these pores are likely to dictate the selective permeability of carboxysome shells (Tsai et al., 2007; Kinney et al., 2011), which are impermeable to CO_2_ (Dou et al., 2008; Cai et al., 2009), and permeable to protons (Menon et al., 2010). α-carboxysomes contain RubisCO and carbonic anhydrase, as described above. Based on amino acid sequence, α-carboxysomal carbonic anhydrase (CsoSCA) was initially believed to be a new form of this enzyme, but its structure clarified that it is a deeply divergent β-carbonic anhydrase (So et al., 2004; Sawaya et al., 2006). α-carboxysomes also contain CsoS2, which facilitates the assembly of these microcompartments by binding to RubisCO and CsoS1 (Cai et al., 2015; Oltrogge et al., 2020). The conserved nature of α-carboxysome shell proteins and contents is reflected in gene synteny apparent in the loci encoding them; typical gene order in α-carboxysome loci is *cbbL*, *cbbS*, *csoS2*, *csoSCA*, *csoS4AB*, and *csoS1ABC*, with *csoS1D* genes, when present, often encoded a few genes downstream or elsewhere (Figure 1; Cannon et al., 2002; Cai et al., 2008; Roberts et al., 2012; Axen et al., 2014; Sutter et al., 2021).

Atypical carboxysome loci are scattered among several phyla of *Bacteria* (Table 1). Most are present in genomes from members of *Proteobacteria*, as expected, given the abundance of organisms from this phylum with typical α-carboxysome loci (Cannon et al., 2002; Axen et al., 2014; Sutter et al., 2021). The atypical loci described here fall into four categories: (1) *csoSCA* is present without any of the other carboxysome-associated genes; (2) *cbbL* and *cbbS* and *csoSCA* are present, without genes encoding shell proteins; (3) genes encoding RubisCO are missing from the locus, with *cbbL* and *cbbS* encoded in a location distinct from *csoS1*, *csoS2*, *csoSCA*, and *csoS4*; and (4) *csoSCA* is absent, though the other carboxysome-associated genes are present (Figure 1). It seems likely that these atypical loci originated from typical loci, and were selected for in some lineages. The objective of this review is to assess the likelihood that the genes of these atypical loci are functional, predict the function of the loci, and describe how they may have originated.

**Table 1:** Number of genomes with atypical carboxysome loci.

Type of locus^a^	*Proteobacteria*			Other phyla
	*Alpha*	*Beta*	*Gamma*	
Just *csoSCA*	2	27	63	28 from eight phyla^b^
*cbbLS* and *csoSCA*	3	2	–	–
No *CsoSCA*	–	4	7	One from *Actinobacteria*
*cbbLS* and *csoS1-4* separate	–	4	–	–

a Atypical carboxysome loci were gathered from the Integrated Microbial Genomes & Microbiomes database (IMG; https://img.jgi.doe.gov; Chen et al., 2019). To find these atypical carboxysome loci, two lists of genomes from isolates were compared: (1) the list of all genomes containing genes encoding members of Pfam12288 (*csoS2*) or Pfam08936 (*csoSCA*), believed to be exclusive to carboxysomes (collected using the “find functions” feature at IMG), and (2) the list of all genomes containing typical α-carboxysome loci, with 10 kb regions of genome sequence encoding members of Pfam00016 (*cbbL*), Pfam00101 (*cbbS*), Pfam00936 (*csoS1*), Pfam12288 (*csoS2*), Pfam08936 (*csoSCA*), and Pfam03319 (*csoS4*) (collected using the “cassette search” feature at IMG). Genomes absent from list (2) were examined more closely to determine whether they had atypical carboxysome loci, or whether genes were absent due to sequencing gaps. To remove carboxysome loci likely to be incomplete due to sequencing gaps, draft genomes, and genomes from this list with >100 scaffolds were removed. For the remainder of the genomes on the list, the gene neighborhoods of the *csoS2* and *csoSCA* genes were examined, and those in which these genes were located at the end of a scaffold were removed. The remaining loci were manually reviewed to verify the presence and absence of *cbbL*, *cbbS*, *csoS1*, *csoS2*, *csoSCA*, and *csoS4*.

b Phyla in which CsoSCA homologs are present outside of carboxysome loci include *Candidatus* Falkowbacteria (eight genomes), *Candidatus* Magasanikbacteria (two genomes), *Candidatus* Moranbacteria (nine genomes), *Candidatus* Pacebacteria (one genome), *Candidatus* Staskawiczbacteria (one genome), *Candidatus* Uhrbacteria (three genomes), *Chrysogenetes* (two genomes), and *Nitrospira* (two genomes).

## Do the Genes From Atypical Carboxysome Loci Encode Functional Proteins?

The majority of genes from atypical carboxysome loci appear to encode proteins that could function similarly to their homologs from typical carboxysome loci, based on the presence of conserved amino acids predicted from their sequences (Table 2). For CbbS sequences from members of genus *Nitrobacter*, conserved residue Y25 (tyrosine) is replaced with histidine; given that both are large polar amino acids, this substitution may not disrupt the function of CbbS in these organisms. *Pseudonocardia* sp. N23 has two *cbbS* genes, with one (IMG gene ID 2868417193) immediately upstream of *cbbL*, and the other (IMG gene ID 2868417191) immediately downstream (Figure 1). The protein encoded by the upstream *cbbS* is only 63 amino acids long, shorter than is typical for CbbS (~90 amino acids), and is missing several conserved amino acids (L53, P54, and F56) in the portion that is present. The protein encoded by the *cbbS* gene downstream *cbbL*, as annotated in IMG, has a truncated amino terminus, but selecting an alternative start codon results in a predicted amino acid sequence including S2, L11, and P12. Based on these observations, the *cbbS* gene downstream of *cbbL* in *Pseudonocardia* sp. N23 is likely to be functional, while the *cbbS* upstream is not.

**Table 2:** Conserved residues in carboxysome-associated proteins.

Protein	Model organism	Conserved amino acids^*^	Function	Reference
CbbL	*Rhodospirillum rubrum* (CbbM)^†^	K166, K191, D193, E194, H287, G393, and G395	Active site residues	Watson et al., 1999
CbbS	*Cupriavidus necator*	S9, L11, P12, Y25, E36, W48, L53, P54, F56, and E67	Nearly universally conserved in CbbS	Spreitzer, 2003
CsoS1A,B,C	*Halothiobacillus neapolitanus*	D25, K29, V36, G37, R51, G52, V57, A63, G64, A67, R70, I80, I81, R83, H85, L92, and P93	Conserved in both α- and β-carboxysomes	Tsai et al., 2007; Kinney et al., 2011
CsoS2	*Halothiobacillus neapolitanus*	N region repeats; M region repeats; and Conserved C terminus		Cai et al., 2015; Oltrogge et al., 2020
CsoSCA (CsoS3)	*Halothiobacillus neapolitanus*	C173, D175, R177, H242, C253, H397, and E399	Active site residues	Sawaya et al., 2006
CsoS4	*Halothiobacillus neapolitanus*	V6, D40, G43, V50, S56, A58, D70, and D/E78	conserved	Zhao et al., 2019

* Conserved amino acids are numbered relative to their position in the amino acid sequence from the model organism.

† CbbM (form II RubisCO) is homologous to CbbL (large subunit of form I RubisCO); both CbbM and CbbL catalyze the carboxylation of ribulose 1,5-bisphosphate (Tabita et al., 2008).

Genes encoding CsoS1A-C from atypical carboxysome loci have some amino acid substitutions at conserved positions. In many cases the substitutions are biochemically similar; e.g., V36 (valine) is replaced with an isoleucine, R70 (arginine) is replaced with a lysine, I80 (isoleucine) is replaced with a valine, which suggests similar functionality. However, there are some instances, e.g., for *Pseudonocardia* sp. N23, where the amino acids are not biochemically similar [V36 (valine) is replaced with glutamate; G37 (glycine) is replaced with aspartate]; given that these are core residues of CsoS1 monomers, folding may be problematic, suggesting that these carboxysomes may not be able to assemble.

Genes encoding CsoS2 from atypical carboxysome loci have features that have been found to be conserved among sequences from typical carboxysome loci. All have at least one N-terminal [RK]XXXXX[HKR]R motif, which binds RubisCO (Cai et al., 2015; Oltrogge et al., 2020). Of the six repetitive motifs from the M (middle) region of CsoS2 (Cai et al., 2015), M1–M4 and M6 are present, while M5 is less conserved. All share a conserved carboxy terminus as described in (Cai et al., 2015).

α-Carboxysomal carbonic anhydrase encoded by atypical carboxysome loci, including those from loci consisting solely of *csoSCA* homologs, have all of the active site residues. In typical carboxysome loci, *csoSCA* follows *csoS2*. Members of genus *Thiomicrospira* have a gene following *csoS2* which in some cases matches weakly with Pfam08936 (see section “No *csoSCA*” below), but lack all of the active site residues necessary for carbonic anhydrase activity.

Amino acid sequences predicted from genes encoding CsoS4A and B from all of the atypical carboxysome loci include all of the conserved residues, though in some cases S56 (serine) is replaced with threonine; given that a hydroxyl moiety is present in both of these amino acids, this substitution is less likely to be disruptive to the function of these proteins.

## Taxonomic Distribution, Origin, and Potential Function of the Four Types Of Atypical Carboxysome Loci

As described above, based on predicted amino acid sequences, most of the individual genes of atypical carboxysome loci appear to encode proteins sufficiently conserved to be capable of the same function as their homologs from typical carboxysome loci. Below are detailed descriptions of the taxonomic distribution of atypical loci, possible mechanisms for their origins, and predictions of how the proteins encoded by atypical carboxysome loci could function together.

### *csoSCA* Alone

**Figure 2:**
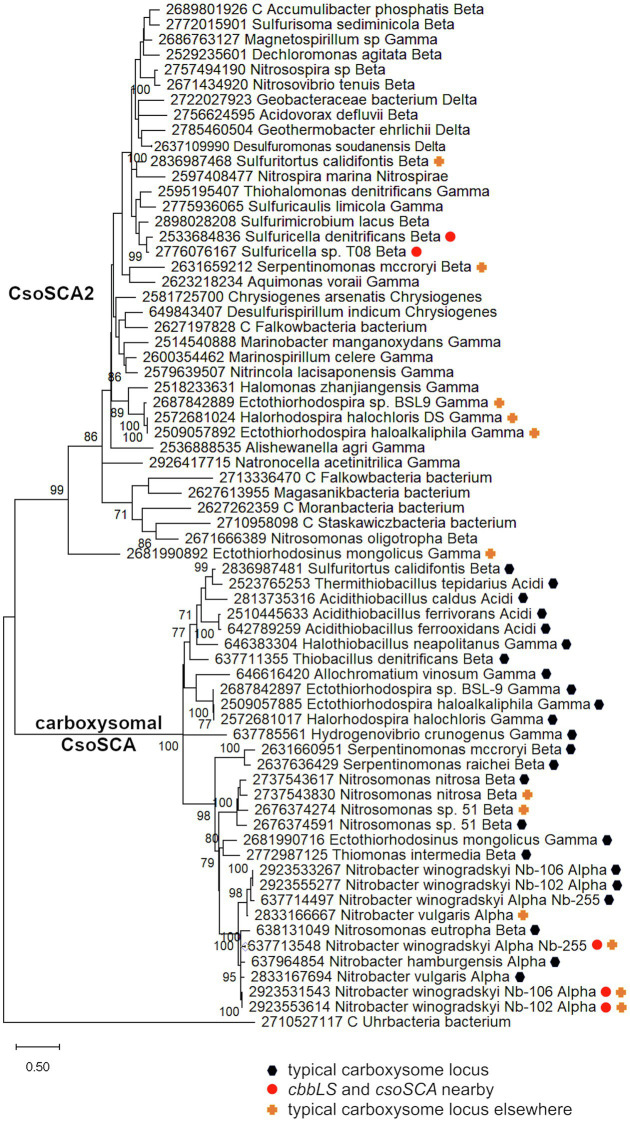
Maximum likelihood analysis of CsoSCA homologs from carboxysome loci and elsewhere (CsoSCA2). Amino acid sequences were gathered from the IMG database, aligned by MUSCLE in MEGA 11, and trimmed *via* GBLOCKS to a final length of 278 aa (Edgar, 2004; Talavera and Castresana, 2007; Tamura et al., 2021). The maximum likelihood tree was constructed with partial deletion of gaps (95% cut-off) and the JTT model (Jones et al., 1992; discrete Gamma distribution with five categories, gamma parameter = 1.9314, 3.55% of sites evolutionarily invariant; this model had the lowest AIC calculated *via* the Find Best DNA/Protein Models feature in MEGA 11; Hurvich and Tsai, 1989; Akaike, 1998). Branch lengths are proportional to the number of substitutions (scale bar = substitutions per site). Bootstrap values are based on 500 resamplings of the alignment, with values <70% omitted. Taxon labels include abbreviated names of classes of *Proteobacteria* (*Alpha*, *Beta*, *Gamma*, and *Delta*; *Acidi* = *Acidithiobacillia*), and full names of phyla beyond *Proteobacteria*. “C” indicates candidate status of species or phylum names. Taxon names also include symbols indicating the position of CsoSCA homologs relative to carboxysome-related genes, if present in the genomes. “Typical carboxysome locus” indicates that the CsoSCA homolog is part of a typical carboxysome locus, “*cbbLS* and *csoSCA* nearby” indicates that genes encoding RubisCO and a CsoSCA homolog are juxtaposed on the genome, and “typical carboxysome locus elsewhere” indicates that a typical carboxysome locus is present elsewhere on the genome.

**Figure 3:**
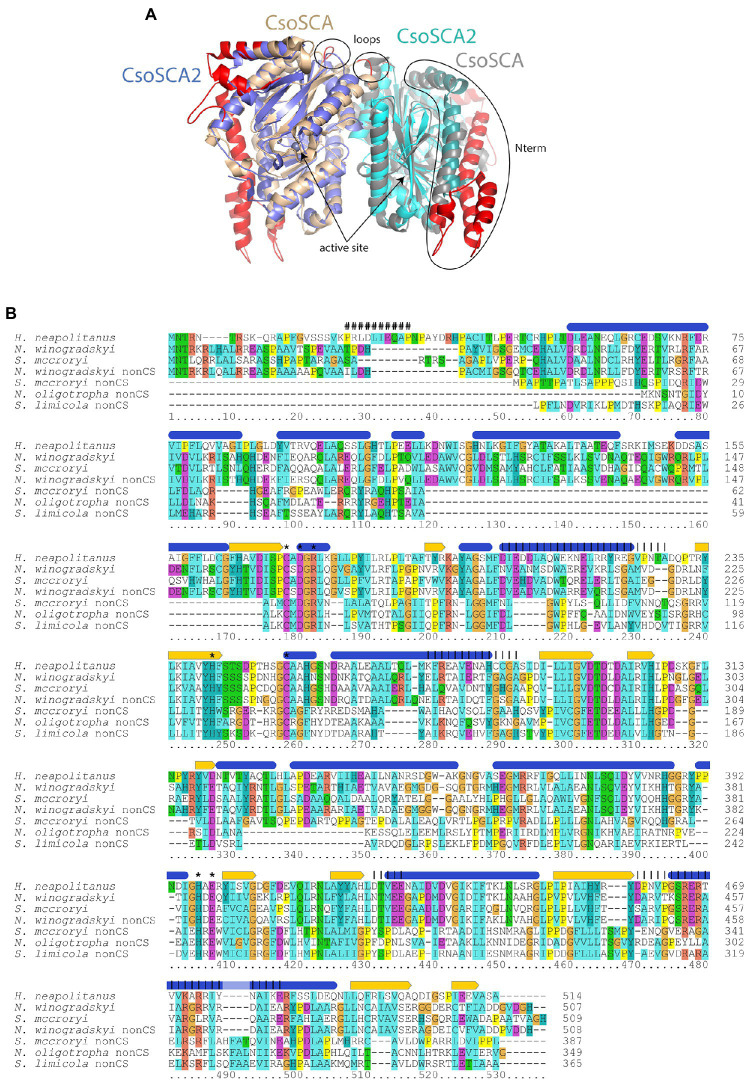
Comparison of carboxysomal CsoSCA and CsoSCA2. **(A)** Cartoon representation (www.pymol.org) of a dimer of CsoSCA subunits (pdb id 2FGY) in gray and wheat, and AlphaFold2 model of CsoSCA2 in blue and cyan. The active site zinc is labeled and shown as a sphere. Differences due to truncations are shown on the CsoSCA in red. The two N-terminal helices of CsoSCA2 are shown as slightly darker blue/cyan. Loop regions with truncations at the dimer interface are also labeled. **(B)** Alignment of carboxysomal CsoSCA and CsoSCA2 sequences. Structural and functional information from *Halothiobacillus neapolitanus* CsoSCA is indicated above the alignment: Blue ovals = alpha-helices, yellow arrows = beta strands, “#” = RubisCO binding site, “*” = active site residue, vertical lines = regions involved in dimerization. Coloring of conserved residues is according to chemical properties. Sequences from typical carboxysome loci included in the alignment are: *Halothiobacillus neapolitanus*, *H. neapolitanus* from *Gammaproteobacteria*, IMG gene object ID 646383304; *Nitrobacter winogradskyi*, *N. winogradskyi* from *Alphaproteobacteria*, IMG gene object ID 2923555277; *Serpentimonas mccroryi*, *S. mccroryi* from *Betaproteobacteria*, IMG gene object ID 2631660951. CsoSCA2 sequences included in the alignment are: *N. winogradskyi* nonCS, *N. winogradskyi* from *Alphaproteobacteria*, IMG gene object ID 2923553614; *Serpentimonas mccroryi* nonCS, *S. mccroryi* from *Betaproteobacteria*, IMG gene object ID 2631659212; *Nitrosomonas oligotropha* nonCS, *N. oligotropha* from *Betaproteobacteria*, IMG gene object ID 2671666389; *Sulfuricaulis limicola* nonCS, *S. limicola* from *Gammaproteobacteria*, IMG gene object ID 2775936065.

Genes homologous to those encoding CsoSCA are quite widespread beyond carboxysome loci, and are present in genomes from autotrophic (e.g., *Sulfuritortus caldifontis*, *Nitrospia marina*) and heterotrophic (e.g., *Cand*. Accumulibacter phosphatis; *Chrysiogenes arsenatis*) bacteria. Given their widespread distribution, it is surprising that they have yet to be studied (Table 1; Figure 2; referred to as *csoSCA2* to distinguish them from those present in carboxysome loci). The amino acid sequences predicted from *csoSCA2* genes share many features with CsoSCA proteins; they include both an active and defunct domain (Sawaya et al., 2006), and the active domain includes all of the residues necessary for catalytic activity as carbonic anhydrase (Table 2; Figure 3).

There are two variants of CsoSCA2. The first variant closely resembles carboxysomal CsoSCA (found in *Nitrobacter vulgaris*, *Nitrobacter winogradskyi*, *Nitrosomonas nitrosa*, and *Nitrosomonas* sp. 51), clustering with carboxysomal CsoSCA in phylogenetic analyses, but missing the N-terminal 40 residues (Figure 2; Figure 3). The second variant is further truncated at the N-terminus, is missing short stretches of sequence throughout, and does not cluster with carboxysomal CsoSCA sequences (Figure 2; Figure 3). The more substantially truncated version has an N-terminal domain of only 40 amino acids (instead of 144 for the CsoSCA from *Halothiobacillus neapolitanus*) that does not align with the CsoSCA equivalent on a sequence level but is also predicted to form two short alpha helices in an AlphaFold2 model (Jumper et al., 2021; Figure 3). Further truncations include shorter loops connecting secondary structure elements (Figure 3). Some of those extra elements are involved in dimer contacts (Sawaya et al., 2006), so it is possible that this homolog has lost the ability to form dimers, which would be unusual for a β-carbonic anhydrase (Cannon et al., 2010); however, this would need to be verified experimentally. The N-terminus of CsoSCA from *Htb. neapolitanus* facilitates interaction between CsoSCA and RubisCO (Blikstad et al., 2021). Presumably, since CsoSCA2 proteins do not interact with RubisCO, this N-terminal region is not necessary for CsoSCA2 to function outside of carboxysomes. Altogether, this form of CsoSCA2 seems to be a more compact version, possibly due to the fact that it is not necessary to encapsulate this protein in a carboxysome. This is particularly interesting for genomes that include both *csoSCA* and *csoSCA2* genes (e.g., members of *Nitrobacter*, *Nitrosomonas*, *Ectothiorhodospira*, and *Halorhodospira*). Presumably, the CsoSCA2 proteins cannot assemble within the carboxysomes present in these organisms.

The presence of CsoSCA2 sequences in numerous phyla, and the more restricted distribution of CsoSCA, suggest that CsoSCA may have originated from CsoSCA2. However, in some cases, the reverse appears to be the case. *Nitrobacter vulgaris*, *Nb. winogradskyi* (Nb-102, 106, and 255), *Ns. nitrosa*, and *Nitrosomonas* sp. 51 have genes encoding both a CsoSCA (encoded in a typical carboyxsome locus), and a CsoSCA2 encoded elsewhere. The two copies cluster together within the larger clade of carboxysomal CsoSCA sequences, despite having the truncated N-termini seen in other CsoSCA2 sequences (Figure 2; Figure 3). Sequence similarities between CsoSCA and CsoSCA2 proteins in these organisms suggest that these CsoSCA2 sequences duplicated and diverged from CsoSCA.

One wonders why these deeply divergent β-carbonic anhydrases are so widespread, and what the features of these proteins might be that make them particularly useful to their host organisms. Though CsoSCA2 proteins lack the residues needed to associate with RubisCO, they may have residues that facilitate the formation of other types of enzyme complexes. Alternatively, CsoSCA2 proteins may not require aggregation with other proteins for activity. Indeed, even carboxysomal CsoSCA is active when expressed in the absence of other carboxysomal proteins (Heinhorst et al., 2006), which suggests that “free” CsoSCA2 could also be active in the cytoplasm of its host organisms.

### *cbbL*, *cbbS*, and *csoSCA* Alone

**Figure 4:**
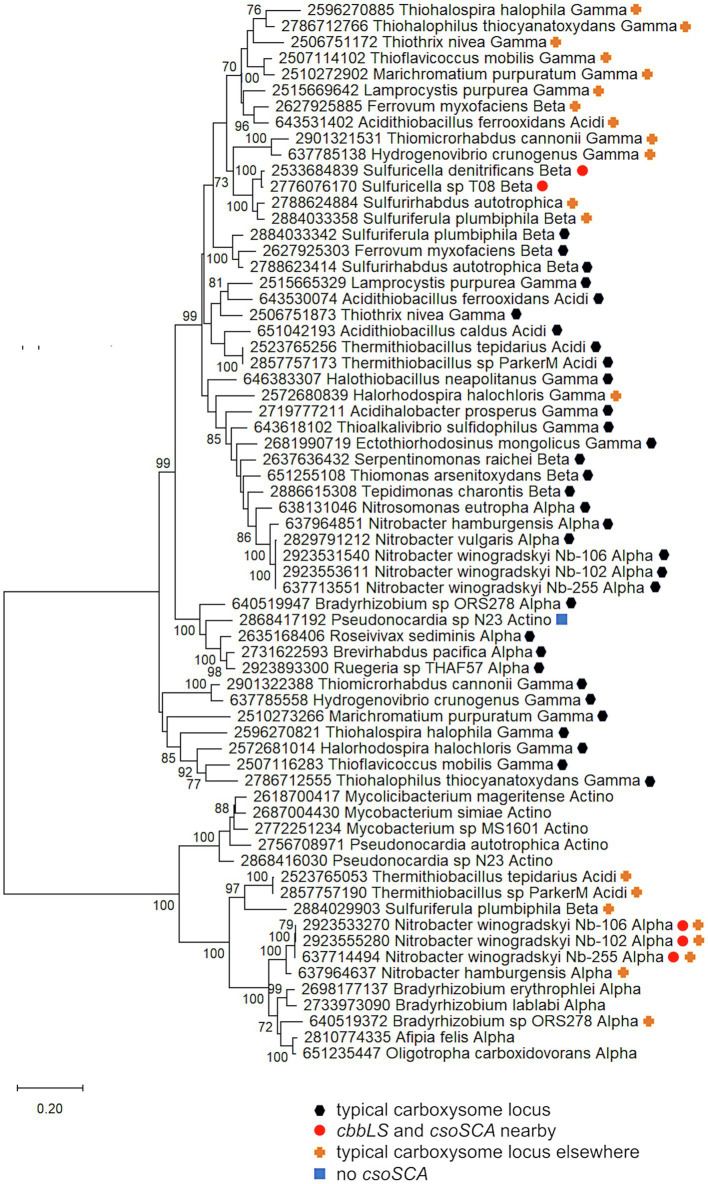
Ribulose 1,5-carboxylase/oxygenase subunits (CbbL and CbbS) encoded by genes collocated with *csoSCA* homologs. Maximum likelihood analysis of CbbL and CbbS sequences was undertaken on sequences gathered from the IMG database, aligned by MUSCLE in MEGA 11, and trimmed *via* GBLOCKS (Edgar, 2004; Talavera and Castresana, 2007; Tamura et al., 2021). CbbL and CbbS alignments were then concatenated using the FASTA alignment joiner feature at FABOX (https://birc.au.dk/~palle/php/fabox/index.php), resulting in an alignment of 527 residues. The tree was constructed with partial deletion of gaps (95% cut-off) and the Le_Gascuel model (Le and Gascuel, 2008; discrete Gamma distribution with five categories, gamma parameter = 0.5775, 17.46% of sites evolutionarily invariant; this model had the lowest AIC calculated *via* the Find Best DNA/Protein Models feature in MEGA 11; Hurvich and Tsai, 1989; Akaike, 1998). Branch lengths are proportional to the number of substitutions (scale bar = substitutions per site). Bootstrap values are based on 500 resamplings of the alignment, with values <70% omitted. Taxon labels include abbreviated names of classes of *Proteobacteria* (*Alpha*, *Beta*, *Gamma*, and *Delta*; *Acidi* = *Acidithiobacillia*), and members of *Actinobacteria* (*Actino*). Taxon names also include symbols indicating the position of *cbbL* and *cbbS* genes relative to carboxysome-related genes, if present in the genomes. “Typical carboxysome locus” indicates that the *cbbL* and *cbbS* genes are part of a typical carboxysome locus, “*cbbLS* and *csoSCA* nearby” indicates that genes encoding RubisCO and a CsoSCA homolog are juxtaposed on the genome, “typical carboxysome locus elsewhere” indicates that a typical carboxysome locus is present elsewhere on the genome, and “No CsoSCA” indicates that the carboxysome locus lacks *csoSCA*.

In genomes from some members of *Alpha*- and *Betaproteobacteria*, *csoSCA* homologs are present near genes encoding RubisCO (Figure 1; Figure 4). In *Alphaproteobacteria*, three strains of *Nb. winogradskyi* share this arrangement of genes (though the average nucleotide identities of strains Nb-102 and Nb-106 versus Nb-255 are 94.6%, suggesting they may be a different species; Richter and Rosselló-Móra, 2009). Among *Betaproteobacteria*, two species of *Sulfuricella* have *cbbL* and *cbbS* genes near *csoSCA* homologs (Figure 4).

For both the *Alphaproteobacteria* and *Betaproteobacteria*, if these genomically juxtaposed *cbbL*, *cbbS*, and *csoSCA* genes are the fragments of a single degraded carboxysome locus, one would anticipate that phylogenetic analysis would place them among genes encoding their carboxysomal cognates from taxonomically affiliated organisms. For the *Nitrobacter* spp. and *Sulfuricella* spp., the *cbbLS* genes cluster with noncarboxysomal RubisCO genes (Figure 4). The situation is more complicated for the *csoSCA* homologs (Figure 2). For the *Nitrobacter* spp., the *csoSCA* homologs appear to be recent duplicates of those present in the typical carboxysome loci in their genomes. For the *Sulfuricella* spp., the *csoSCA* homologs fall within the *csoSCA2* clade and are unlikely to have arisen from carboxysome loci. Given the noncarboxysomal origin of the *cbbLS* genes in both classes, and *csoSCA* gene in the *Sulfuricella* spp., these are not fragments of a single degraded carboxysome locus.

Despite the likelihood that they do not share evolutionary history cohabitating carboxysomes, it is still possible that these two enzymes might function together in the cytoplasm to facilitate CO_2_ fixation in their host organisms, all of which are capable of autotrophic growth (Winogradsky, 1892; Kojima and Fukui, 2010). Coregulation is possible for both, but seems more likely for the members of *Nitrobacter*, since their genes are <2 kb apart (Figure 1). The juxtaposition of noncarboxysomal RubisCO genes to those encoding typical β-carbonic anhydrase has been noted for two members of *Hydrogenovibrio* (Yoshizawa et al., 2004; Scott et al., 2006), and is apparent in genome data from in many other members of *Hydrogenovibrio* and *Thiomicrorhabdus*,^1^ suggesting such juxtaposition may be selected for in some organisms. While the expression of cytoplasmic carbonic anhydrase results in a high CO_2_-requiring phenotype in organisms with CCMs (Price and Badger, 1989), there is evidence for carbonic anhydrase activity in the chloroplasts of certain algae and plants (reviewed in Moroney et al., 2001). If these enzymes do function together in *Nitrobacter* and *Sulfuricella*, perhaps the carbonic anhydrase facilitates RubisCO-mediated CO_2_ fixation by maintaining intracellular HCO_3_^−^ and CO_2_ near chemical equilibrium, preventing RubisCO from diminishing the concentration of intracellular CO_2_ under conditions where CCMs are not induced (e.g., moderate environmental CO_2_ concentrations; Yoshizawa et al., 2004).

### *cbbL* and *cbbS* Separate From *csoS1*, *csoS2*, *csoSCA*, and *csoS4*

Many members of family *Thiobacillaceae* (Boden et al., 2017; Boden, 2019) have *csoS1*–*S4* genes in a separate genomic locus from *cbbL* and *cbbS* genes (Figure 1), as has previously been described for *Thiobacillus denitrificans* (Cannon et al., 2003; Beller et al., 2006a). Of the eight genome sequences from cultivated members of this family, all of which grow autotrophically (Boden et al., 2017; Boden, 2019), five include a homolog to *csoS2* (Pfam012288; *Annwoodia aquaesulis*, *Sulfuritortus calidifontis* DSM103923 and J1A, *Thiobacillus denitrificans* ATCC25259, and *Thiobacillus thioparus*). In all cases, these *csoS2* genes do not have *cbbL* and *cbbS* genes immediately upstream. Instead, *cbbL* and *cbbS* are located 2.6–21 kb away from *csoS1-4* (Figure 1). The other three genome sequences lack *csoS2* homologs; since these three sequences are incomplete (38–98 scaffolds), it is not possible to know whether *csoS2* is truly absent from these organisms. Nine genomes (15–407 scaffolds) inferred to belong to members of *Thiobacillaceae* have been gathered from metagenomes, and five of these include *csoS2* homologs. Three of these genes are present at the ends of scaffolds, making it impossible to determine whether *cbbL* and *cbbS* genes are nearby. For the two remaining (*Thiobacillus* spp. Bin4_E1B and BP01), *cbbL* and *cbbS* are encoded separately from *csoS1*–*4*. Based on these observations, it seems likely that having *csoS1*–*4* genes apart from *cbbL* and *cbbS* genes may be a trait shared by all members of this family.

There are two mechanisms that could have resulted in the separate *cbbLS* and *csoS1-4* loci found in members of *Thiobacillaceae*. In the first scenario, a typical ancestral carboxysome locus containing all of these genes was severed by genome re-arrangement. In the second scenario, *cbbLS* and *csoS1-4* did not share an ancestral locus. Instead, carboxysomal *cbbLS* genes could have been lost from the genome entirely, and the *cbbLS* genes currently located 2–20 kb away are noncarboxysomal in origin. An additional possibility is that either *cbbLS* or *csoS1-4* were acquired *via* horizontal gene transfer.

**Figure 5:**
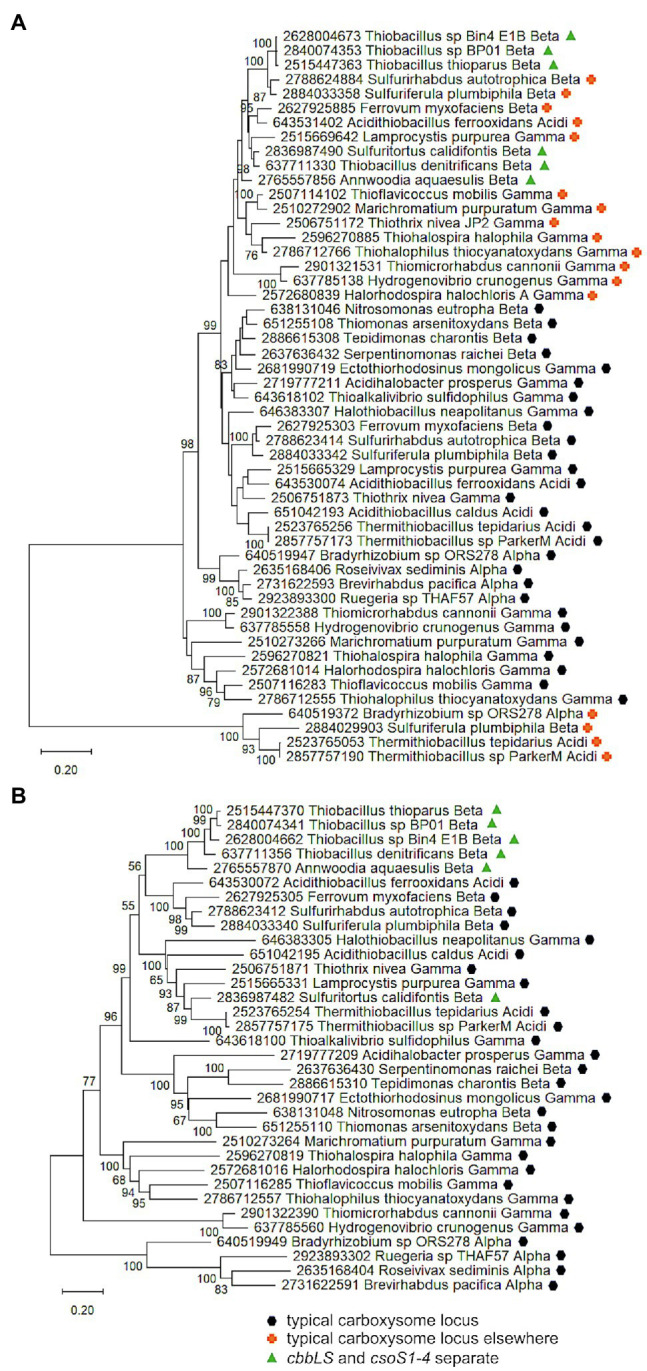
Analysis of RubisCO subunits (CbbL and CbbS) and carboxysome shell proteins (CsoS2, SCA, S4A, and S4B) that are encoded at two separate loci. Maximum likelihood analysis of amino acid sequences of **(A)**. RubisCO subunits and **(B)**. carboxysome shell proteins was undertaken on sequences that were gathered from the IMG database, aligned by MUSCLE in MEGA 11, and trimmed *via* GBLOCKS (Edgar, 2004; Talavera and Castresana, 2007; Tamura et al., 2021). CbbL and CbbS alignments were then concatenated using the FASTA alignment joiner feature at FABOX (https://birc.au.dk/~palle/php/fabox/index.php), as were CsoS2, SCA, S4A, and S4B, resulting in alignments of 550 (CbbLS) and 997 (CsoS2–4) residues. The trees were constructed with partial deletion of gaps (95% cut-off) and the Le_Gascuel model [Le and Gascuel, 2008; discrete Gamma distribution with five categories, gamma parameter = 0.5298, 17.64% of sites evolutionarily invariant (CbbLS); gamma parameter = 1.2921, 7.86% of sites evolutionarily invariant (CsoS2-4); this model had the lowest AIC calculated *via* the Find Best DNA/Protein Models feature in MEGA 11; Hurvich and Tsai, 1989; Akaike, 1998]. Branch lengths are proportional to the number of substitutions (scale bar = substitutions per site). Bootstrap values are based on 500 resamplings of the alignment, with values <70% omitted. Taxon labels include abbreviated names of classes of *Proteobacteria* (*Alpha*, *Beta*, *Gamma*, and *Delta*; *Acidi* = *Acidithiobacillia*). Taxon names also include symbols indicating the position of genes relative to carboxysome-related genes, if present in the genomes. “Typical carboxysome locus” indicates that the genes are part of a typical carboxysome locus, “*cbbLS* and *csoS1-4* separate” indicates that genes encoding RubisCO and CsoS1–4 are encoded by separate loci, and “typical carboxysome locus elsewhere” indicates that a typical carboxysome locus is present elsewhere on the genome.

To provide evidence for different mechanisms for formation of *cbbLS* and *csoS1-4* loci, phylogenetic analyses were conducted using concatenated alignments of *cbbL* and *cbbS* genes (*cbbLS*), and *csoS2*, *S3*, *S4a*, and *S4b* (*csoS2-4*). Genes encoding CsoS1A-C were omitted from these analyses, due to difficulties distinguishing the three types of *csoS1A-C* genes. The results of these analyses raise the possibility that *cbbLS* and *csoS1-4* loci did not originate from a single ancestral typical carboxysome locus in these organisms (Figure 5). In *Sf. calidifontis* (here collapsed to strain J1A, since sequences for the two strains are identical), *cbbLS* genes fall in a small well-supported clade with *Tb. denitrificans*, and with other members of *Thiobacillaceae* in larger clades, though these larger clades are not as well-supported (Figure 5). The *csoS2-4* genes from *S. calidifontis* fall among completely different taxa than its *cbbLS* genes, suggesting independent origins for its two loci. For all four isolates from *Thiobacillaceae*, *cbbLS* genes do not fall among those from typical carboxysome loci, though it should be noted that carboxysomal and non-carboxysomal *cbbLS* genes are not distinguished by two distinct, well-supported clades (Figure 5). Together, these observations suggest independent origins for *cbbLS* and *csoS2-4* loci in *Thiobacillaceae*, but low bootstrap values for these phylogenetic analyses compromise the confidence of this assertion. If the *cbbL* and *cbbS* genes in members of *Thiobacillaceae* did not originate from a typical carboxysome locus, it would be very interesting to verify that they were capable of being packed into carboxysomes, as thus far it seems that noncarboxysomal RubisCO from other organisms cannot be packed into carboxysomes (Menon et al., 2008).

Currently, evidence for the presence of carboxysomes in members of *Thiobacillaceae* is limited to *Tb. thioparus*. Transmission electron micrographs have only been published for *Tb. thioparus* and *Tb. denitrificans*; polyhedral inclusions are apparent in *Tb. thioparus* cells, but not in *Tb. denitrificans* (Shively et al., 1970). Given the synteny of the carboxysome loci among *Thiobacillus* sp. Bin4 E1B, *Thiobacillus* sp. BP01, and *Tb. thioparus*, as well as the placement of their *cbbLS* and *csoS2-4* genes together in clades (Figure 4), it seems likely that all three of these organisms are capable of synthesizing carboxysomes. For *Tb. denitrificans*, the absence of carboxysomes despite the presence of *csoS1-4* is puzzling, and cannot be attributed to strain-level differences, since both ultrastructure and genome sequence were obtained from the same strain (ATCC25259). Perhaps their synthesis could be induced under growth conditions different from those used to cultivate the cells for ultrastructural study.

If carboxysomes are indeed synthesized by these organisms, one possible advantage of having separate loci would be independent regulation of *cbbLS* and *csoS1-4* loci. Transcriptome analysis of *Tb. denitrificans* is consistent with this possibility. Based on hybridization with microarrays, transcripts of the *Tb. denitrificans cbbL* and *cbbS* genes are particularly abundant under aerobic conditions, but no such changes are apparent for *csoS1-4* (Beller et al., 2006b). Other organisms have two sets of *cbbLS*, with one in a typical carboxysome locus and the other located elsewhere on the genome. These organisms synthesize noncarboxysomal RubisCO when CO_2_ concentrations are moderate, and selectively synthesize carboxysomal RubisCO and shell proteins when CO_2_ concentrations are very low (Yoshizawa et al., 2004). Perhaps some members of *Thiobacillaceae* upregulate the *cbbLS* locus when CO_2_ concentrations are low to moderate, and reserve upregulation of the *csoS1-4* locus for low CO_2_ conditions, or other circumstances where carboxysomes facilitate growth.

### No *csoSCA*

**Figure 6:**
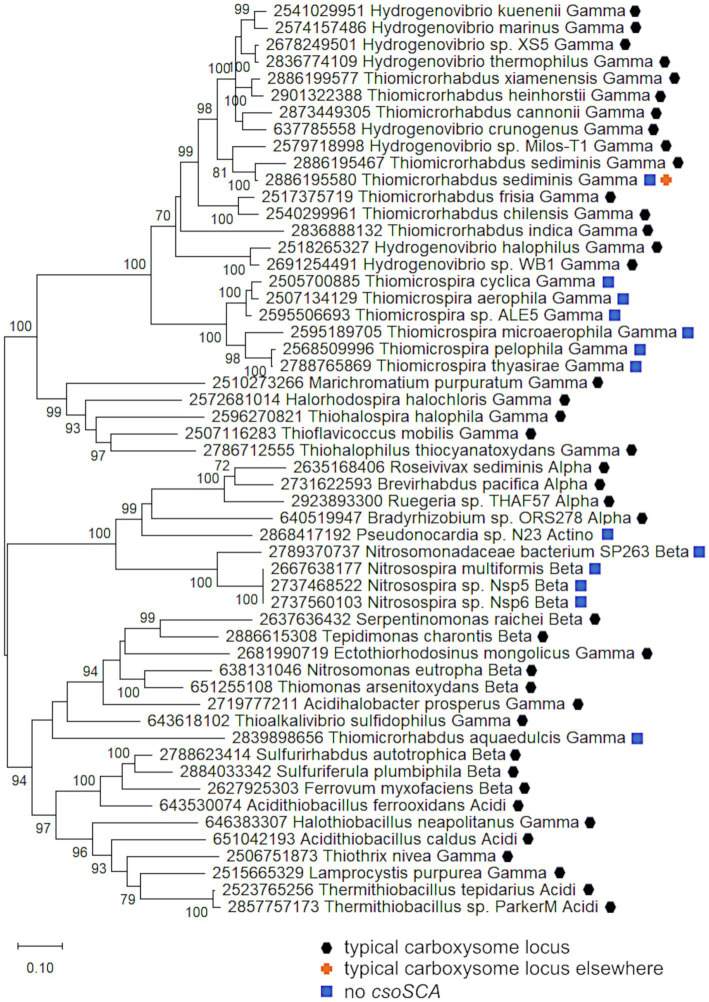
Carboxysome loci lacking *csoSCA* genes. Maximum likelihood analysis was undertaken on amino acid sequences of RubisCO subunits (CbbL and CbbS) and carboxysome shell proteins (CsoS2, S4A, and S4B) gathered from the IMG database, aligned by MUSCLE in MEGA 11, and trimmed *via* GBLOCKS (Edgar, 2004; Talavera and Castresana, 2007; Tamura et al., 2021). CbbL, CbbS, CsoS2, CsoS4A, and CsoS4B alignments were then concatenated using the FASTA alignment joiner feature at FABOX (https://birc.au.dk/~palle/php/fabox/index.php), resulting in an alignment of 940 residues. The trees were constructed with partial deletion of gaps (95% cut-off) and the Le_Gascuel model (Le and Gascuel, 2008; discrete Gamma distribution with five categories, gamma parameter = 0.7037, 16.40% of sites evolutionarily invariant; this model had the lowest AIC calculated *via* the Find Best DNA/Protein Models feature in MEGA 11; Hurvich and Tsai, 1989; Akaike, 1998). Branch lengths are proportional to the number of substitutions (scale bar = substitutions per site). Bootstrap values are based on 500 resamplings of the alignment, with values <70% omitted. Taxon labels include abbreviated names of classes of *Proteobacteria* (*Alpha*, *Beta*, *Gamma*, and *Delta*; *Acidi* = *Acidithiobacillia*) and members of *Actinobacteria* (*Actino*). Taxon names also include symbols indicating the position of genes relative to carboxysome-related genes, if present in the genomes. “Typical carboxysome locus” indicates that the genes are part of a typical carboxysome locus, “typical carboxysome locus elsewhere” indicates that a typical carboxysome locus is present elsewhere on the genome, and “No CsoSCA” indicates that the carboxysome locus lacks *csoSCA*.

Carboxysome loci lacking *csoSCA* genes arose multiple times; they are present in some autotrophic organisms from *Beta*- and *Gammaproteobacteria*. A carboxysome locus lacking *csoSCA* is also present in *Pseudonocardia* sp. N23, a member *Actinobacteria* (Figure 1); though it has not been determined whether this organism could grow autotrophically, other members of its genus can (e.g., *Pseudonocardia autotrophica*; Takamiya and Tubaki, 1956). None of these organisms have *csoSCA* homologs elsewhere in their genomes (aside from *Thiomicrorhabdus sediminis*, which has a copy in its “typical” carboxysome operon). In *Betaproteobacteria*, they are present in *Nitrosospira muliformis* and also *Nitrosospira* spp. Nsp5 and Nsp6, which may be strains of *Nsp. multiformis*, based on average nucleotide identities >99% (Richter and Rosselló-Móra, 2009). Within *Gammaproteobacteria*, they appear to have arisen independently three times. All members of *Thiomicrospira* have carboxysome loci lacking *csoSCA*. Within *Thiomicrorhabdus*, such loci seem to have arisen twice. In *Thiomicrorhabdus sediminis*, two carboxysome loci are present; one is typical, while the second appears to be a recent duplicate of the typical locus. The amino acid sequences predicted from both *cbbL* and *cbbS* genes are 100% identical. Both copies of CsoS2 are 100% identical at amino termini; however, at residue 330, they diverge, and this continues to the carboxy termini. CsoS1 sequences also are identical at the amino termini and have small differences at their carboxy termini. The carboxysome locus from *Thiomicrorhabdus aquaedulcis* does not fall within a clade with those from other members of its genus (Figure 6), suggesting that it may have been acquired *via* horizontal gene transfer.

The carboxysome locus from *Pseudonocardia* sp. N23 includes *cbbL* and *cbbS* genes distinct from those present in other members of phylum *Actinobacteria* (Figure 4). Other members of this phylum carry *cbbL* and *cbbS*, and *Pseudonocardia* sp. N23 does include a copy that falls within a clade of these sequences (Figure 4). However, the *cbbL* and *cbbS* genes present in the carboxysome locus, as well as *csoS2*, *csoS4A*, and *csoS4B*, fall among genes from carboxysome loci from members of *Alpha*- and *Betaproteobacteria* (Figures 4, 6), suggesting this locus was acquired *via* horizontal gene transfer.

It is apparent that these carboxysome loci originated from typical carboxysome loci, given that they cluster with others that contain *csoSCA* genes (Figure 6). Indeed, in members of *Thiomicrospira*, a gene is present between *csoS2* and *csoS4A* (Figure 1), which is likely to be a degraded form of *csoSCA*. In *Thiomicrospiras pelophila*, *thyasirae*, and *microaerophila*, these genes do match Pfam08936 (*csoSCA*), but e-values range from 0.006 to 4.5e−05, and none of the residues necessary for carbonic anhydrase activity are present. However, the amino termini of the proteins predicted from these genes align well with those from CsoSCA proteins. Given that the amino termini of CsoSCA proteins may facilitate interactions among carboxysome proteins (Blikstad et al., 2021), perhaps these degraded genes may still encode proteins that facilitate packing of RubisCO molecules into carboxysomes.

There is evidence that these carboxysome loci are transcribed and translated. Carboxysome locus genes are transcribed in *Tms. pelophila* (Scott et al., 2019), carboxysomes are visible in transmission electron micrographs of members of *Thiomicrospira* (Sorokin et al., 2001, 2002a,b; Scott et al., 2019), and have been purified from *Tms. thyasirae* (Lanaras et al., 1991). Electron dark inclusions are abundant in *Nsp. muliformis*, but staining patterns suggest these consist of glycogen (Watson et al., 1971). Ultrastructural studies of *Pseudonocardia* sp. N23, as well as *Tmr. aquadulcis* and *sediminis*, have not been published.

Given the presence of carboxysomes in at least some of these taxa, the conservation of residues necessary for the function of the CbbL, CbbS, CsoS2, and CsoS4A and B proteins, and the convergent evolution of this sort of carboxysome locus in multiple lineages of microorganisms, they are likely to be functional in their host organisms (however, see the comments on CsoS1 sequences from *Pseudonocardia* sp. N23 in Section “Do the Genes From Atypical Carboxysome Loci Encode Functional Proteins” above). The current understanding of carboxysome function requires the presence of carbonic anhydrase activity within these microcompartments in order for them to facilitate CO_2_ fixation by RubisCO (see above). One possibility is that these modified carboxysomes have shells that are permeable to CO_2_, allowing this gas to enter from the cytoplasm. CsoS4 proteins are necessary for carboxysome shell impermeability to CO_2_; the absence of CsoS4 to seal the vertices of their shells renders the microcompartments CO_2_-permeable (Cai et al., 2009). Their critical function perhaps accounts for their strong sequence conservation, hence redundancy, which is unusual for bacterial microcompartments that have multiple pentamer-forming paralogs (Melnicki et al., 2021). Interestingly, the carboxysome locus from *Tmr. aquaedulcis* lacks genes encoding CsoS4A, and the *Tmr. sediminis* locus lacking *csoSCA* lacks both *csoS4A* and *csoS4B*. Perhaps carboxysomes from these organisms operate without CsoS4 proteins, and are permeable to CO_2_. Given that carboxysome shells are assumed to require only 12 pentamers, and their pores are small (~4 Å in diameter), they are assumed to not play a significant role in metabolite conductance. However, a recent study of the protein stoichiometry of β-carboxysomes showed varying occupation of the vertices by the CcmL, the lone pentamer-forming gene product in beta carboxysome loci (Sun et al., 2019). The occupancy was correlated with environmental conditions, suggesting that pentamer association with shells is dynamic and perhaps serves as one way to alter permeability. Because *Tmr. sediminis* has two carboxysome loci (one typical, one lacking *csoSCA*, *csoS4A*, and *csoS4B*), determining the conditions under which it expresses typical, vs. atypical, carboxysomes could provide useful information about how its atypical carboxysomes might function, including whether pentamers and carbonic anhydrase are provided by the other locus. If these carboxysomes are permeable to CO_2_, cytoplasmic CO_2_ concentrations would need to be elevated in order to enhance RubisCO activity, running the risk of high rates of CO_2_ loss from the cells *via* diffusion, unless this loss is counterbalanced by living in a high CO_2_ habitat. These organisms have been cultivated in growth media supplemented with HCO_3_^−^ (10–30 mM; Kojima and Fukui, 2019) or CO_2_ (20% headspace; Liu et al., 2021). For *Tmr. sediminis*, the lack of *csoS4* genes suggests that this organism may not be capable of growth under low CO_2_ conditions, and it would be interesting to determine whether this is the case.

The other organisms lacking CsoSCA have loci including genes encoding CsoS1 and CsoS4; perhaps their shells are permeable to CO_2_ based on modifications to these two types of shell proteins. However, such differences are not detected when shell proteins from typical carboxysomes are compared to those from carboxysomes lacking carbonic anhydrase. For CsoS1ABC proteins, the sequence FVGGGY, corresponding to residues 40–45 from *Htb. neapolitanus*, comprises the narrowest part of the pore and the residues surrounding it (Tsai et al., 2007). In all of the CsoS1ABC sequences from atypical carboxysome loci lacking *csoSCA*, these residues are conserved, suggesting the pores have characteristics similar to those in typical carboxysomes. To determine whether there are other residues that vary systematically for these atypical carboxysome loci, and to detect changes in the sequence that are more likely due to the presence/absence of CsoSCA rather than evolutionary distance, CsoS1ABC sequences within *Piscirickettsiaceae* were compared, since genomes from this family include both typical (all 10 species of *Hydrogenovibrio*, 8/10 species from *Thiomicrorhabdus*) and atypical (2/10 species from *Thiomicrorhabdus*, all six members of *Thiomicrospira*) loci. Among all of these organisms, CsoS1ABC sequences are highly conserved throughout the sequences. Likewise, CsoS1D sequences from these organisms have small differences throughout, and mapping those differences on a homology model does not reveal significant patches of variability. CsoS4A and B sequences are also very similar across all three genera and there are no distinguishable differences between them. If these shell proteins actually are permeable to CO_2_, the mechanism mediating this change is not apparent from their sequences.

Another mechanism for preserving the activities of these carboxysomes would be their recruitment of a carbonic anhydrase encoded elsewhere on the genome, as may be the case for some β-carboxysomes from *Cyanobacteria*. β-carboxysomes carry homologs to γ-carbonic anhydrase (Dearaujo et al., 2014). In some cases, these homologs are enzymatically active as carbonic anhydrases, while in others, these homologs have apparently lost enzymatic activity (Cot et al., 2008), although the active site residues are intact. In these cases, the carboxysomes also carry a functional β-carbonic anhydrase (deeply divergent to CsoSCA; So et al., 2002; Cot et al., 2008; Rae et al., 2013), and the gene encoding this β-carbonic anhydrase is not present in or near the operon encoding the essential components of the carboxysome (Rae et al., 2013). Evaluation of these possibilities awaits purification of carboxysomes from organisms with carboxysome loci lacking *csoSCA* genes, to test the permeabilities of their shells and the potential presence of carbonic anhydrase activity within them.

## Conclusion

The unusual carboxysome-related loci described here are common enough to suggest relevance. Genes encoding CsoSCA2 are extremely widespread. Colocalization of *csoSCA* homologs and *cbbLS* is present in genomes from two classes of *Proteobacteria*. “Split” carboxysome loci (*cbbLS* and *csoS1-4*) are likely present in all members of family *Thiobacillaceae*. Carboxysome loci lacking *csoSCA* homologs (or homologs unlikely to be active) are present in at least two classes of *Proteobacteria* and have been horizontally transferred to phylum *Actinobacteria*. Together, all of this indicates that modified carboxysome loci have been evolutionarily selected for in some lineages, and are not the tattered remnants of typical carboxysome loci, captured on their journey to degradation and loss. Understanding how the proteins encoded by these atypical carboxysome loci function could help us understand better how typical carboxysomes function (the exceptions that prove—or disprove—the rule), as well as the selective pressures driving their origins from the assembly of their components over time.

The nature of the selective advantage provided by these atypical loci is not apparent at this point. All of the organisms carrying these atypical carboxysome loci (except for *csoSCA2*) are chemolithoautotrophs, so these atypical loci are likely to play a role in CO_2_ fixation. The habitats from which they were isolated are very diverse, with CO_2_ concentrations ranging from extremely low (alkaline soda lakes; Sorokin et al., 2001, 2002a,b), to high (e.g., soils, marine sediments; Bock and Wagner, 2006; Kelly and Wood, 2006). Particularly for those organisms from low CO_2_ habitats, these atypical carboxysome loci are likely to play a role in CCMs. Consistent with this possibility, most of these organisms have genes for likely DIC transporters either associated with their atypical loci or elsewhere in their genomes (Scott et al., 2020). However, based on the current understanding of CCMs in bacteria, which requires that both RubisCO and carbonic anhydrase are present in carboxysomes, it is difficult to understand how organisms lacking carboxysomal RubisCO (as in section “*cbbL* and *cbbS* Separate From *csoS1*, *csoS2*, *csoSCA*, and *csoS4*” above) or carbonic anhydrase (as in section “No *csoSCA*” above) could have functioning CCMs. This conceptual gap may result from the relative paucity of studies on CCMs in organisms besides *Cyanobacteria*, in which CCMs have been well-studied (reviewed in Price et al., 2009). Though carboxysomes from chemolithoautotrophs have been well-studied (Kerfeld et al., 2010, 2018; Sutter et al., 2021), their integration with the other components of CCMs in these organisms (e.g., DIC transporters) has not. CCM function (carboxysome presence and elevated intracellular DIC concentration) has been demonstrated for only one bacterium beyond *Cyanobacteria* (*Hydrogenovibrio crunogenus*; Dobrinski et al., 2005). Upregulation of genes encoding both DIC transporters and carboxysomes under low DIC conditions has only been demonstrated for a handful of chemolithoautotrophic *Gammaproteobacteria* (Mangiapia et al., 2017; Desmarais et al., 2019; Scott et al., 2019). Despite this undersampling, it is already apparent that CCMs in *Proteobacteria* are more diverse than those from *Cyanobacteria*, in their reliance on a different arsenal of DIC transporters and multiple types of RubisCO (Dobrinski et al., 2012; Scott et al., 2019, 2020). Atypical carboxysomes could represent yet another layer of diversity in these CCMs; evaluating this possibility awaits further study of CCMs in these organisms as well as those in other members of *Proteobacteria*.

## Author Contributions

KS contributed to conception and design of the manuscript. KS, USF GC 2020, USF GC 2021, MS, and CK ran the analyses. All authors contributed to manuscript writing and revision, and have read and approved the submitted version.

## Funding

This work was supported by the National Science Foundation (NSF-MCB-1952676 to KS), and the Office of Science of the United States Department of Energy (DE-FG02-91ER20021 to MS and CK), and MSU AgBio Research (MS and CK).

## Conflict of Interest

The authors declare that the research was conducted in the absence of any commercial or financial relationships that could be construed as a potential conflict of interest.

## Publisher’s Note

All claims expressed in this article are solely those of the authors and do not necessarily represent those of their affiliated organizations, or those of the publisher, the editors and the reviewers. Any product that may be evaluated in this article, or claim that may be made by its manufacturer, is not guaranteed or endorsed by the publisher.

## Footnotes

^1^ https://img.jgi.doe.gov/
