# Corrigendum: Atypical carboxysome loci: JEEPs or junk?

**DOI:** 10.3389/fmicb.2024.1393362

**Published:** 2024-04-04

**Authors:** USF Genomics Class 2020, USF Genomics Class 2021, Markus Sutter, Cheryl A. Kerfeld, Kathleen M. Scott

**Affiliations:** ^1^Integrative Biology Department, University of South Florida, Tampa, FL, United States; ^2^MSU-DOE Plant Research Laboratory, Michigan State University, East Lansing, MI, United States; ^3^Environmental Genomics and Systems Biology and Molecular Biophysics and Integrated Bioimaging Divisions, Lawrence Berkeley National Laboratory, Berkeley, CA, United States

**Keywords:** carboxysome, microcompartment, carbonic anhydrase, carbon dioxide fixation, autotroph

In the published article, there was an error. A correction has been made to the **Acknowledgments**. This section previously stated:

The authors are grateful to the University of South Florida for the resources to make it possible for the students in Genomics Class 2020 and 2021 to contribute to this manuscript. Genomics Class 2020 and 2021 are Salvador Ayala-Preciado, Alizain K. Bangash, Eleanor Brodrick, Deanna Byram, Madilyn Carney, Michael Colosi, Holly David, Gabriela Dolard, Anelys Figueroa, Christyn A. Geniesse, Lucy B. Gravitt, M. Andrew Glass, Rhiannon A. Hendrick, Arianna Henry, Thomas Henry, Alexis Hunt, Nicole Juern, Jason A. Kalathia, Taylor Kelsay, Jacob Klingman, Sydney T. Marshall, Ian M. Matosdeaza, Aaron S. Mohammed, Jeremias Munoz, Nicole Nauman, Dhanvi Patel, Alexandra Rodier, Beatriz Sanchez Uribazo, Logan Suits, Angel D. Valladares, Daniela Varon-Garcia, Alex Wagner, Lauren Wallace, and Jana Wieschollek.

The corrected section appears below:

We are grateful to the University of South Florida for the resources to make it possible for the students in Genomics Class 2020 and 2021 to contribute to this manuscript. Genomics Class 2020 and 2021 are Salvador Ayala-Preciado, Alizain K. Bangash, Eleanor Brodrick, Deanna Byram, Madilyn Carney, Michael Colosi, Holly David, Gabriela Dolard, Anelys Figueroa, Christyn A. Geniesse, Lucy B. Gravitt, M. Andrew Glass, Rhiannon A. Hendrick, Arianna Henry, Thomas Henry, Alexis Hunt, Nicole Juern, Jason A. Kalathia, Taylor Kelsay, Jacob Kligman, Sydney T. Marshall, Ian M. Matosdeaza, Aaron S. Mohammed, Jeremias Munoz, Nicole Nauman, Dhanvi Patel, Alexandra Rodier, Beatriz Sanchez Uribazo, Logan Suits, Angel D. Valladares, Daniela Varon-Garcia, Alex Wagner, Lauren Wallace, and Jana Wieschollek.

The authors apologize for this error and state that this does not change the scientific conclusions of the article in any way. The original article has been updated.

